# Ventricular Septal Rupture and Artificial Intelligence (AI)-Assisted Healthcare

**DOI:** 10.7759/cureus.36581

**Published:** 2023-03-23

**Authors:** Aditya Lal Vallath, Barath Prashanth Sivasubramanian, Aryapriyo Chatterjee, Snigdha Erva, Diviya Bharathi Ravikumar, Indraneel Dasgupta

**Affiliations:** 1 Emergency Medicine, Peerless Hospital and B. K. Roy Research Centre, Kolkata, IND; 2 Internal Medicine, Employees State Insurance Corporation Medical College & Employees State Insurance Post Graduate Institute of Medical Sciences and Research (ESIC MC & PGIMSR), Chennai, IND; 3 Internal Medicine, MNR (M. N. Raju) Medical College and Hospital, Fasalwadi, IND

**Keywords:** point-of-care ultrasound (pocus), cardiogenic shock, myocardial infarction, ventricular septal rupture, chatgpt

## Abstract

This case report highlights the use of point-of-care ultrasound (POCUS) for the diagnosis of ventricular septal rupture (VSR), a severe consequence of acute myocardial infarction (AMI). VSR has a broad spectrum of signs and inconspicuous symptoms, making the diagnosis difficult. POCUS offers non-invasive, real-time cardiac imaging and has an advantage over other methods due to its ability to identify VSR early. Here we present a 63-year-old female with a history of type 2 diabetes, hypothyroidism, hyperlipidemia, and a family history of cardiovascular disease, who came to the ED with chest pain for three days, palpitations, and dyspnea at rest. On examination, the patient was hypotensive, tachycardic, and had crackles with a harsh holosystolic murmur. An EKG and elevated troponin levels suggested acute on chronic anterior-lateral wall ST-elevation myocardial infarction (STEMI). Resuscitation efforts were initiated, followed by a lung ultrasound that revealed good lung sliding and multiple B lines without pleural thickening, indicating pulmonary edema. Echocardiography revealed ischemic heart disease with moderate left ventricle (LV) systolic dysfunction and a 14 mm apical ventricular septal rupture (hypokinetic thinning of the anterior wall, septum, apex, and anterolateral wall with a left ventricular ejection fraction (LVEF) of 39%). The presence of flow on color Doppler across the interventricular septum, showing left-to-right shunting, led to a definitive diagnosis of acute-on-chronic myocardial infarction (MI) with ventricular septal rupture. The case report also emphasizes how modern AI applications like ChatGPT (OpenAI, San Francisco, California, United States), aid in language and research, saving time and redefining the healthcare and research industry. As a result, we are confident that AI-assisted healthcare will be the next global breakthrough.

## Introduction

Acute myocardial Infarction (AMI) is a leading cause of mortality and morbidity worldwide [1,2]. Mechanical complications are one of the most severe complications of AMI, which occur in approximately 0.27% of patients with ST-elevation myocardial infarction (STEMI) and 0.06% in those with non-STEMI [1]. Among these complications, ventricular septal rupture (VSR) is one of the most devastating and has a mortality rate of 60-70% [3,4]. The incidence of VSR is rare, but it is associated with increased morbidity and mortality rates [3,4]. The diagnosis of VSR can be difficult because the symptoms of VSR are non-specific, and the signs of VSR can be subtle, such as a new or worsening mitral regurgitation. This can make the diagnosis challenging, particularly in the early stages of the disease [5,6]. The prognosis for patients with VSR is poor, with a median survival of less than a week [3,4].

VSR can occur as a result of infarction in the left ventricle's anterior, inferior, or septal wall. The involvement of the left anterior descending artery is particularly associated with VSR. The clinical features of VSR include hypotension, cardiac shock, and decreased cardiac output [3,4]. Diagnostic tools for VSR include angiography and point-of-care ultrasound (POCUS). POCUS has been shown to be a valuable tool for the diagnosis of VSR, as it is a non-invasive, bedside method that can provide real-time imaging of the heart. POCUS can detect VSR earlier than other modalities and helps to confirm the diagnosis and guide the treatment [7,8].

The purpose of this case report is to present a patient with VSR complicating an acute on chronic myocardial infarction and to discuss the use of point-of-care ultrasound in the diagnosis of VSR. Additionally, this case report has been written with the assistance of ChatGPT, a large language model developed by OpenAI (San Francisco, California, United States), and highlights the utility of advanced artificial intelligence (AI) tools like ChatGPT in assisting authors with the research and writing process.

## Case presentation

A 63-year-old female presented to the emergency department (ED) of a tertiary care hospital with complaints of chest pain for the past three days and shortness of breath and palpitations for the past six hours. The patient denied any symptoms of syncope and had no reported previous cardiac history. The patient belonged to low socio-economic status and had a history of type 2 diabetes mellitus, hypothyroidism, and hyperlipidemia. They had poor compliance with all oral medications and lifestyle advice. The patient was a non-smoker and non-alcoholic. She was not immunized against coronavirus disease 2019 (COVID-19). A family history of ischemic heart disease was noted. On examination, the patient had a Glasgow Coma Scale score of E3, V3, and M5, vital signs showed a systolic blood pressure of 50 mmHg and an unrecordable diastolic blood pressure, tachycardia of 105 beats per minute with regular rhythm and volume, tachypnea with 25 breaths per minute, oxygen saturation of 88% noted in room air.

Upon examination, jugular venous distension was not visible and non-pitting pedal edema was present. On auscultation of the chest, S1 and S2 heart sounds were heard with a rough holosystolic murmur of Grade 4, bilateral air entry was reduced in all lung fields, and coarse crackles were noticed. Immediate resuscitation was achieved by initiating an IV fluid bolus of 500ml of normal saline through a peripheral line; this resulted in her blood pressure increasing to 70/40 mmHg indicating fluid responsiveness. Moist oxygen was started at 4L per minute achieving saturation of 97%. The patient was started on a vasopressor infusion of noradrenaline at 10 mcg/minute. The dosage was titrated to maintain a pressure above 90/60 mmHg. In acute coronary syndrome, loading was achieved with doses of aspirin 325 mg orally (PO), clopidogrel 300 mg PO, and atorvastatin 80 mg PO.

**Figure 1:**
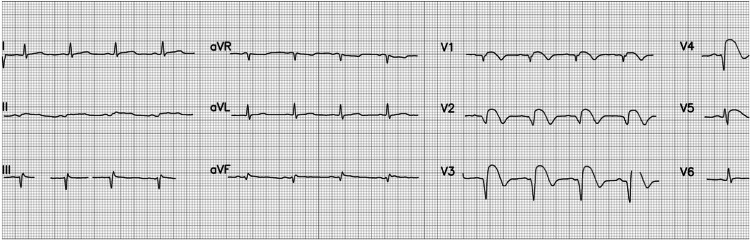
ECG showing acute ST-elevation myocardial infarction Rate 84 beats per minute; Normal sinus rhythm; Normal P wave; Evolving QS waves in leads V2 and V3; ST elevation (Tomb stoning sign) in leads V2 through V5

A 12-lead electrocardiogram was performed and a bedside cardiac troponin test was ordered. The electrocardiogram (Figure 1) showed suspicion of acute STEMI. Based on the EKG findings, involvement of the left anterior descending (LAD) and left circumflex artery was assumed. 

The rapid card troponin I test was performed using a whole blood specimen and was found to be elevated (>100ng/L). As resuscitation efforts were being done, a POCUS scan was performed at the bedside to evaluate cardiac function and evaluate the cause of dyspnea. The procedure revealed multiple B lines (>5) in each of the lung zones bilaterally. Normal lung sliding was noted in all lung zones ruling out pneumothorax. Pleural effusion was not noted and there was no lung thickening, which ruled out pneumonia. An echocardiogram (ECHO) was then performed, which showed an anterior wall, whole septum, apex, and anterolateral wall hypokinetic thinning out. The left ventricular ejection fraction (LVEF) was 39%, with a 14 mm apical VSR, normal valves, and pericardium. An impression of ischemic heart disease, moderate left ventricular systolic dysfunction, and 14 mm apical VSR was noted. Table 1 shows the values recorded using echocardiography.

**Table 1:** Echocardiography Findings

Left Anterior Descending (artery) (LAD)	29 mm.
Ascending Aortic Diameter (AOD)	26 mm.
Aortic Valve Disease Severity (AVDS)	13 mm.
Interventricular Septal Thickness in Diastole (IVSD)	08 mm.
Left Ventricular Posterior Wall Thickness in Diastole (LVPWD)	10 mm
Left Ventricular Internal Diameter in Diastole (LVIDD)	49 mm
Left Ventricular Internal Diameter in Systole (LVIDS)	40 mm
Interventricular Septal Thickness in Systole (IVSS)	11 mm
Left Ventricular Posterior Wall Thickness in Systole (LVPWS)	15 mm
Ejection Fraction	39%
End Systolic Volume (ES)	19%
E-point to Septal Separation (EPSS)	10 mm
Deceleration Time of Early Diastolic Filling Velocity (DE)	16 mm
Ejection Fraction Slope	102 mm/sec
Aortic Forward Velocity	1.17 m/s
Pulmonary Forward Velocity	0.89 m/s

**Figure 2:**
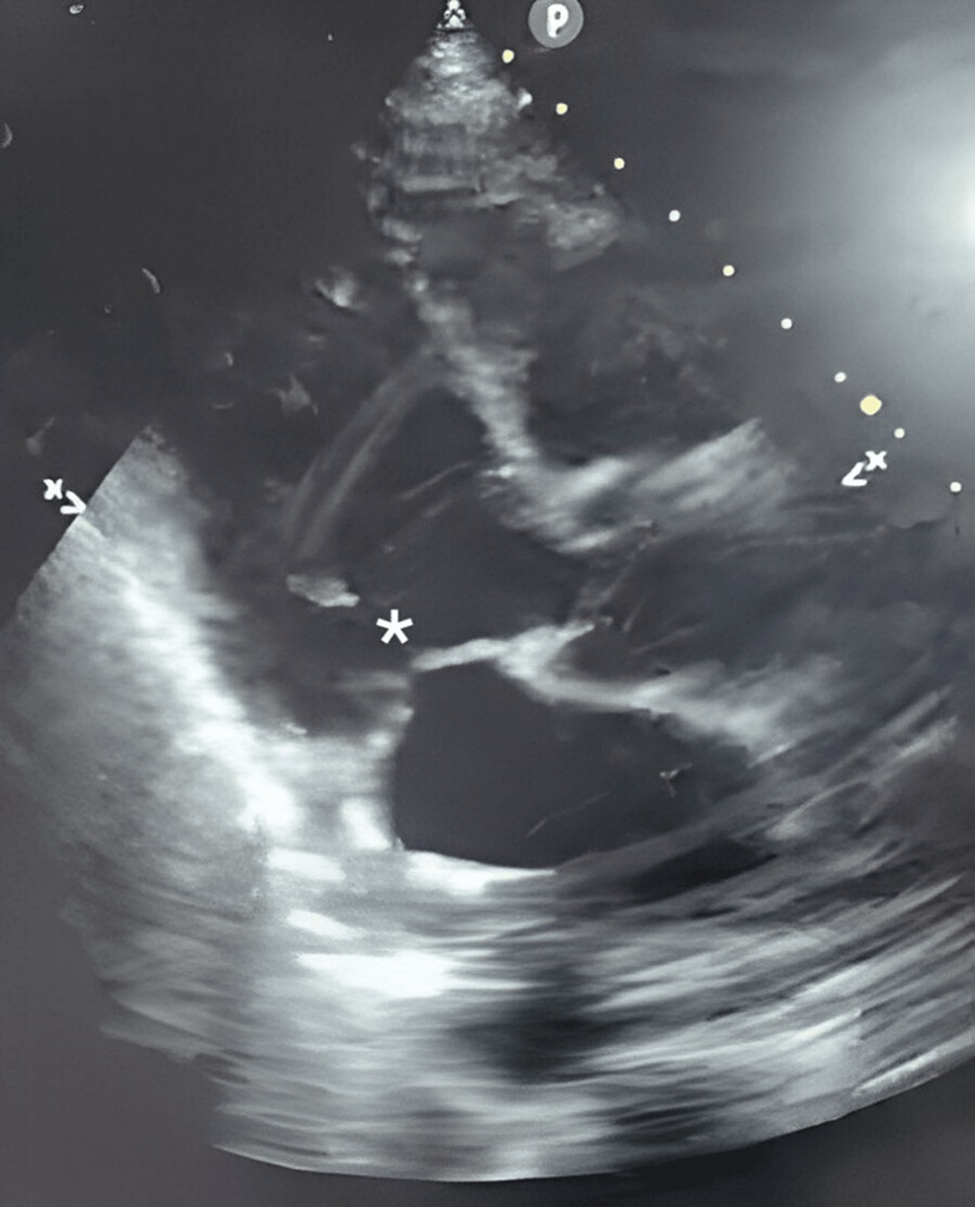
Ultrasound images showing apical ventricular septal rupture * Septal rupture is visible here

**Figure 3:**
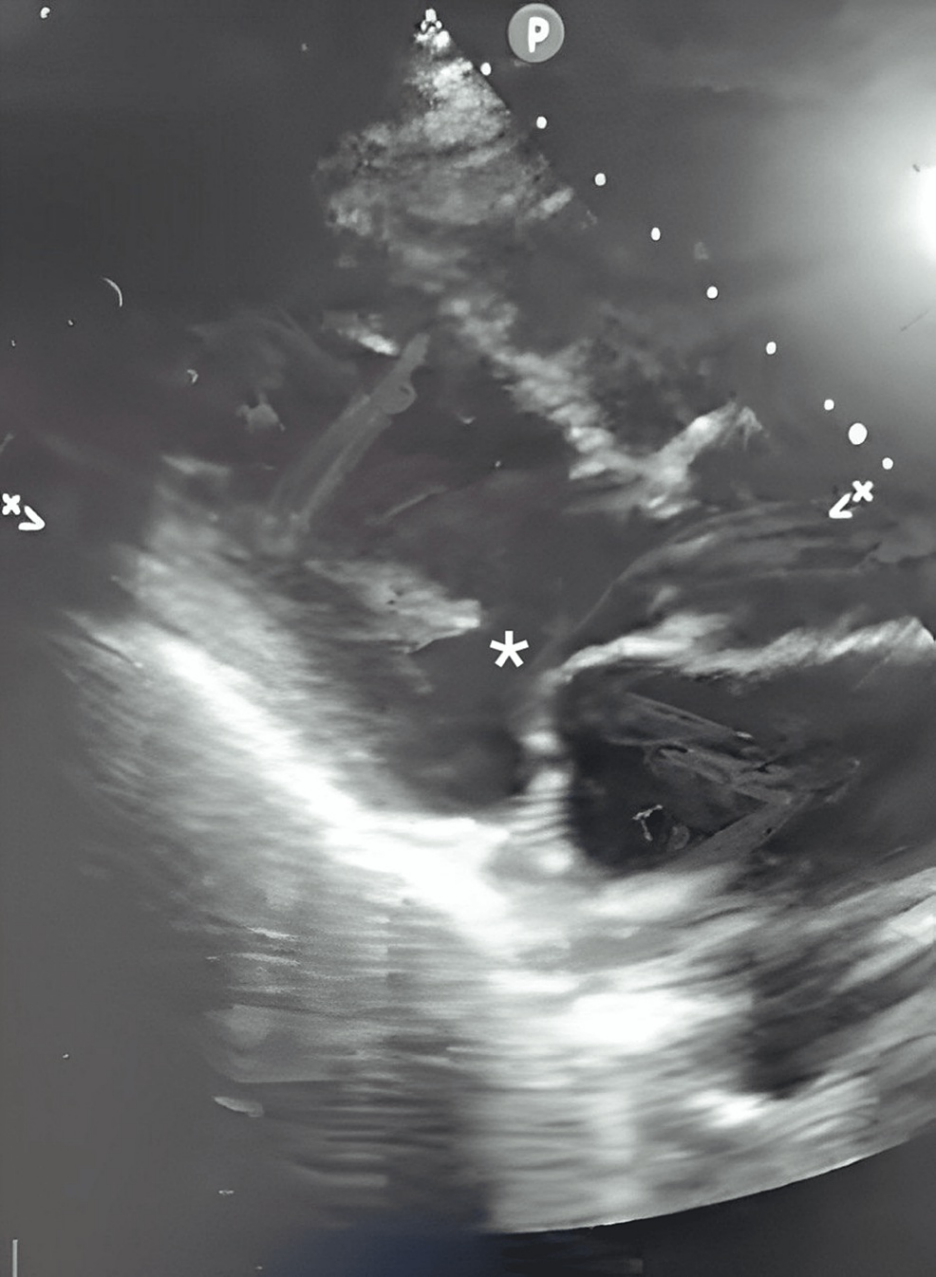
Ultrasound images showing apical ventricular septal rupture * Septal rupture is visible here

**Figure 4:**
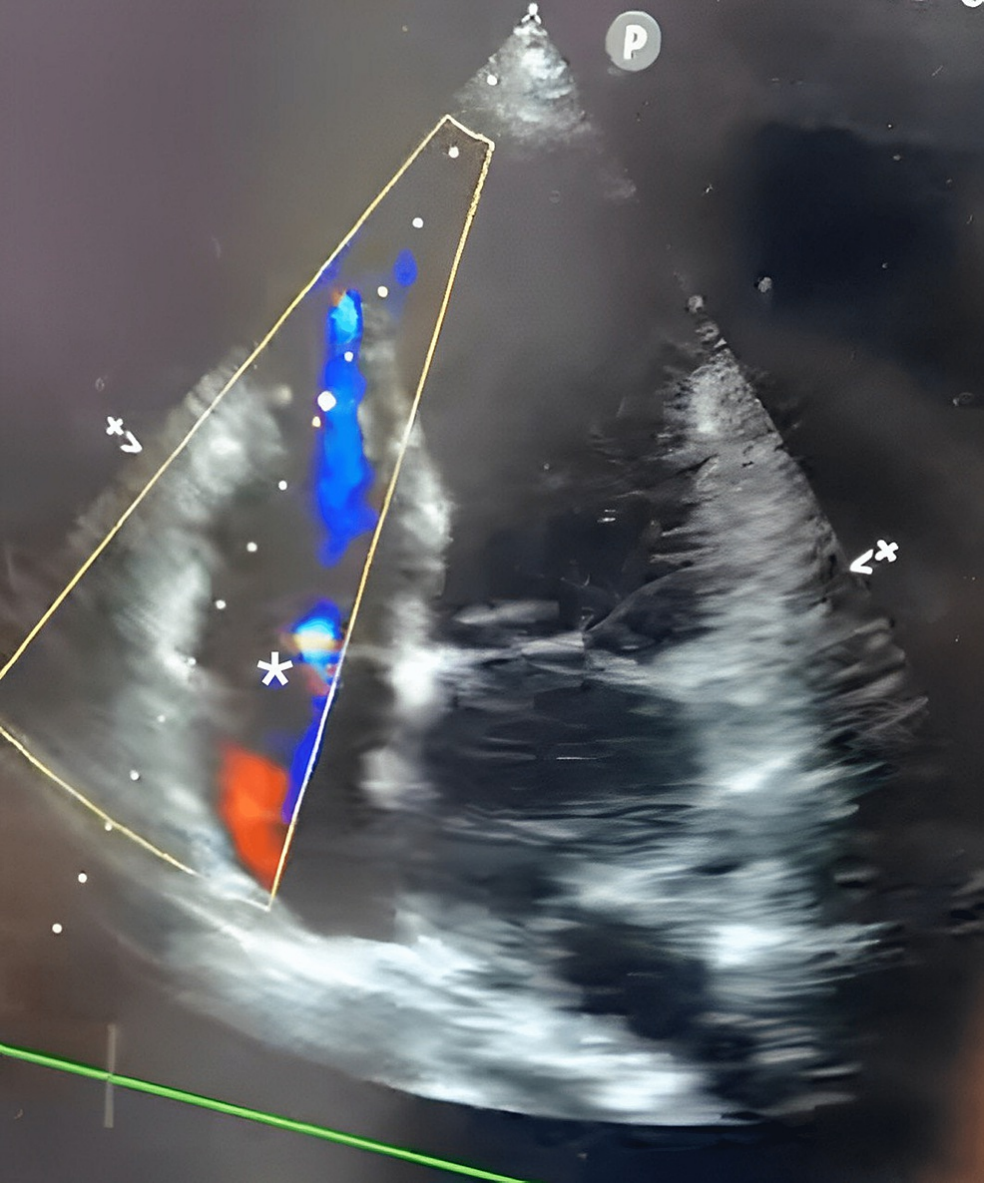
A presence of flow is seen on color Doppler across the interventricular septum from the left to the right ventricle * marks the region of interest

A presence of flow on color Doppler across the interventricular septum from the left to the right ventricle. This provided strong evidence of left-to-right shunting at the ventricular apex. Figures 2-4 show ultrasound and Doppler images.

The patient was diagnosed with acute MI with VSR causing cardiogenic shock. The patient denied further treatment and was discharged following the stabilization of vital signs. 

## Discussion

A 63-year-old female presented to the ED with shortness of breath and palpitations for six hours, along with chest pain for the past 72 hours, and no prior history of cardiac disease. The patient was hemodynamically unstable with an oxygen saturation of 88% on room air. An EKG revealed acute STEMI with involvement of the LAD and left circumflex artery. This is indicative of anterior localization of the infarct, but it is not definitive. POCUS confirmed a VSR with left-to-right shunting at the ventricular apex. Other parameters, such as the echocardiogram and ejection fraction values, were correlated to arrive at the final diagnosis of acute-on-chronic MI with VSR. The patient declined treatment, which would have involved urgent catheterization and repair surgery.

The ventricular septum rupturing after a MI is a severe and often deadly complication that occurs in less than 1% of the population. The survival rate varies greatly depending on the chosen course of treatment: elective surgery performed within 24 hours of the rupture results in a 60% mortality rate, while if done more than 21 days after the rupture, it drops to 10%. On the other hand, if conservative management is opted for, the mortality rate rises to 94% [9]. The pathophysiology of VSR post-MI involves full-thickness (transmural) infarction of the ventricular septum due to coagulation necrosis of ischemic tissue with neutrophil infiltration, resulting in a thinner and weaker septal myocardium [10]. The conventional timing is most likely attributed to the sub-acute phase, which occurs three to five days post-MI [10]. Infarctions caused by right coronary artery (RCA) and LAD occlusion lead to basal defects, while anterior infarctions caused by LAD occlusion result in apical defects. Regardless of the location, the newly established communication causes a left-to-right shunt of oxygenated blood from the high-pressure left ventricle (LV) to the lower-pressure right ventricle (RV) [11]. Based on the EKG findings, involvement of the LAD and left circumflex artery is assumed [11]. Patients with no prior history of angina or MI, ST-segment elevation and signs of Q wave development on the initial EKG, and high MB-creatine kinase levels are 9.2 times more likely to rupture than other patients [12]. Patients with pre-existing coronary artery disease, postoperative renal failure, and a residual postoperative shunt have a worse chance of long-term survival after surviving the immediate postoperative period [13]. 

A meta-analysis of seven prospective cohort studies reported that the use of ECHO in the ED in patients with acute pulmonary edema and bedside critical care echocardiography (CCE) showing B-lines had a high sensitivity (94%) and specificity (92%) [14]. Severe ischemia produces regional wall motion abnormalities (RWMAs) that can be visualized echocardiographically within seconds of coronary artery occlusion and this has been shown to have a high sensitivity (97%) [15-17]. During an AMI, preservation of normal wall thickness and normal reflectivity is seen, and a thin akinetic reflective segment suggests chronicity [18]. Based on similar ECHO findings, we believe the patient had pre-existing coronary artery disease which led to VSR.

In the ED, POCUS is a quick, non-invasive imaging technique that is simple to use and very effective in diagnosing VSR. It is an efficient method for determining the cause of different shock states and other mechanical problems (e.g., RV wall rupture and aortic dissection) in urgent care [19]. Profuse bilateral B-lines with smooth pleural morphology seen during POCUS are indicative of pulmonary edema [20]. According to a study by Hochman et al., patients who underwent revascularization procedures like thrombolytic therapy, intra-aortic balloon counter-pulsation, coronary angiography, angioplasty, or coronary artery bypass surgery had low odds (0.30) of mortality compared to those without the procedure [5]. It was found that patients who did not experience shock following VSR before surgery had a much better chance of survival (82%) compared to those who did (27%) [21]. For high-risk, hemodynamically unstable VSR and cardiogenic shock, the recommended treatment is initial percutaneous coronary intervention (PCI) of the infarct-related artery, followed by immediate surgical closure of the ventricular septal rupture. This approach is expected to quickly establish blood flow and reduce the risks, time, and complexity of the urgent cardiosurgical intervention [22].

According to Jones et al, delaying the procedure is not an option for many individuals as it increases the risk of heart failure and organ dysfunction [11]. Extracorporeal membrane oxygenation devices have been used to stabilize patients before surgery in some case studies [11]. PCI may show single-vessel disease but left ventricular damage and aneurysm formations have been observed in patients with VSR [23]. The medical management of VSR involves afterload reduction using IV sodium nitroprusside to increase effective LV stroke volume and decrease left-to-right shunting. The patient's prognosis depends on the size of the rupture. Minor, hemodynamically stable septal rupture has a favorable prognosis [11]. Table 2 summarizes current evidence of VSR.

**Table 2:** Literature Review LAD: left anterior descending artery; VSR: ventricular septal rupture; PCI: percutaneous coronary intervention; POCUS: point of care ultrasound; PMH: past medical history; MI: myocardial infarction

Author	Year	Patient population	Symptoms and signs	Intervention performed
Bachini et al. [24]	2022	A 57-year-old woman.	Severe shortness of breath and chest pain for seven hours.	Coronary angiography had revealed a thrombotic blockage of the LAD artery, for which PCI and an intra-aortic balloon pump were performed.
Kirbos et al. [25]	2022	A 73-year-old man with a history of hypertension and diabetes mellitus.	Intermittent chest pain for 48 hours that had since become constant, associated with breathlessness and diaphoresis.	A massive ventricular septal rupture with a new ventricular septal defect and concomitant left-to-right shunting was discovered using POCUS.
Wang et al. [26]	2019	A 77-year-old man with a history of MI	Acute onset of breathlessness and palpitations	Echo images revealed a weakening of the interventricular septum close to the apex, where an 8 mm-diameter rupture was observed. It was possible to do interventional occlusion of the ruptured interventricular septum under echocardiography as the patient could not afford thoracotomy.
Portuguese et al. [19]	2020	A 61-year-old man with 45 pack-years smoking history	Complains of chest pain from 1 week and sudden onset dyspnea	A loud holosystolic murmur that persisted throughout the examination increased the possibility of VSR, and POCUS was performed. It confirmed left-to-right flow and exhibited a significant VSR. For definitive management, a delayed surgical VSR repair with concurrent coronary artery bypass grafting was performed.
Dulai et al. [27]	2016	A 59-year-old woman.	Symptoms of acute pulmonary edema and cardiogenic shock 35 days after anterior ST elevation myocardial infarction.	VSR was detected via echocardiography. She underwent a successful ventricular septal defect repair after being immediately transported to the neighborhood cardiothoracic unit.
Michelis et al. [28]	2019	72-year-old male patient, smoker (47 pack-years) with PMH of MI.	Fatigue, shortness of breath for the past 2 months, and bilateral lower limb edema for 2 weeks.	Transthoracic echo revealed a basal inferior and inferoseptal segment aneurysm in the left ventricle, along with right ventricular enlargement, systolic dysfunction, and hypokinesia on the free wall. Due to his stable hemodynamic condition, it was decided to postpone treatment for the septal rupture.
Duraes Campos et al. [29]	2020	A 78-year-old man.	Three weeks before admission, the patient reported an episode of severe chest pain lasting several hours, with limiting and progressive dyspnoea on exertion.	Transthoracic echocardiography revealed a dilated and dysfunctional left ventricle with akinesia of the basal half of the inferior and posterior walls. A large (14 mm), strongly delineated VSR with a turbulent left-to-right shunt at the posterodorsal septum level was confirmed. The patient underwent an urgent heart catheterization, and an intra-aortic balloon pump placement and was referred for emergency surgery.
Delafield et al. [30]	2017	A 56-year-old active male.	Vague symptoms of fatigue, and shortness of breath. His electrocardiogram demonstrated inferior lead ST segment elevation in addition to new q-wave formation.	A large inferior ventricular septal rupture with substantial right ventricular enlargement was discovered during intra-procedural echocardiography. The patient required an intra-aortic balloon pump placed for hemodynamic support and a right ventricular impelled device for right ventricular support and bridge to coronary artery bypass and graft (CABG) with VSR.
Haddar et al. [31]	2022	A 64-year-old patient.	Jaundice with ST-segment elevation MI.	Echocardiography confirmed the diagnosis of a VSR with a left-right shunt and a significant dilation of the right ventricle. VSR was surgically treated, but the outcome was fatal.
Parikh et al. [32]	2020	A 67-year-old male.	Five days of epigastric pressure and dyspnea. He was diaphoretic with mottled extremities.	Coronary angiography revealed right coronary artery blockage, while echocardiography verified a VSR. Venoarterial extracorporeal membrane oxygenation was initiated. Due to progressive multiorgan failure, surgical and percutaneous VSR repairs were deemed futile.

Use of ChatGPT

**Figure 5:**
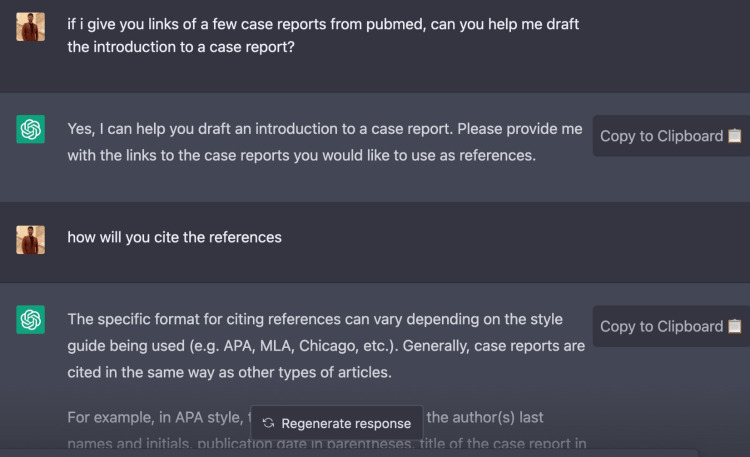
ChatGPT usage in this case report

**Figure 6:**
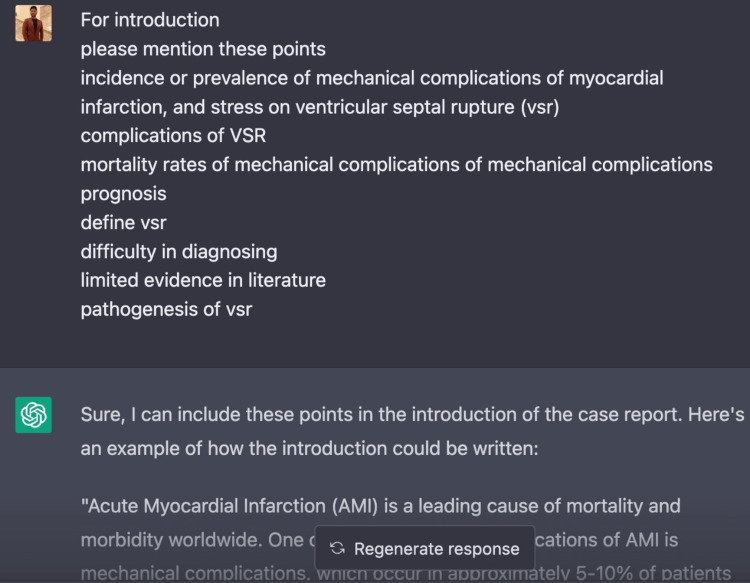
ChatGPT usage in this case report

**Figure 7:**
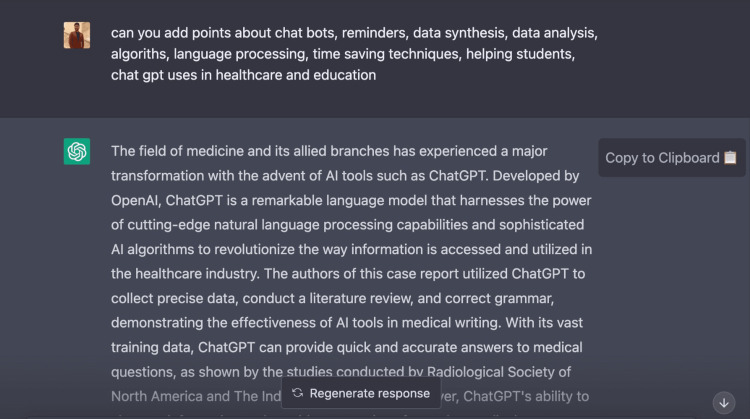
ChatGPT usage in this case report

The field of medicine and its allied branches have experienced a major transformation with the advent of AI tools such as ChatGPT. Developed by OpenAI, ChatGPT is a remarkable language model that harnesses the power of cutting-edge natural language processing capabilities and sophisticated AI algorithms to revolutionize the way information is accessed and utilized in the healthcare industry. The authors of this case report utilized ChatGPT to collect precise data, conduct a literature review, and correct grammar, demonstrating the effectiveness of AI tools in medical writing. With its vast training data, ChatGPT can provide quick and accurate answers to medical questions [33,34]. Moreover, ChatGPT's ability to abstract information and provide summaries of complex medical reports not only saves time but also advances the healthcare industry [35]. In addition to its uses in medical writing, ChatGPT also has applications in data synthesis and analysis, where it can assist in the interpretation of large and complex medical datasets. Its sophisticated algorithms and language processing capabilities enable it to provide reminders to healthcare professionals, helping them stay on top of their schedules and tasks [36]. ChatGPT can also help students in the healthcare field by providing quick and accurate answers to their questions, thus making education more efficient and accessible [37]. Furthermore, chatbots powered by ChatGPT can also play a crucial role in improving patient care by providing quick and relevant information and support [38]. Figures 5-7 describe the usage of ChatGPT in this case report. 

There are limitations to using ChatGPT for research writing, including the fact that it sources information from the internet, but it does not plagiarize it. Because ChatGPT sources its information from the internet's vast database without specific attribution, there is a risk of inherent bias. Therefore, it may be more appropriate to use ChatGPT as an auxiliary tool rather than a primary resource for data extraction. We have identified instances where the core meaning of the information was not conveyed accurately. Therefore, prior knowledge of the topic at hand is necessary while drafting using ChatGPT. We have also found instances where the author names and et al. in the text of the article were cited with names not belonging to the article. So we suggest proofreading the citations for any research writing.

## Conclusions

This case report highlights the use of POCUS for the diagnosis of VSR. The rapid utilization and convenience of use make this a suitable technique to perform at the ED. Following diagnosis, it is appropriate to undergo the necessary interventions. The authors strongly believe that the integration of AI tools such as ChatGPT into the healthcare industry is the next frontier in healthcare innovation. With its remarkable natural language processing abilities, sophisticated algorithms, and vast training data, ChatGPT has the potential to transform the way healthcare professionals work, conduct research, and provide patient care. The authors hope that this case report highlights the immense potential of AI in the field of healthcare and education.
